# Evaluation of a novel Cardiac Peri-Operative Transfusion Trigger Scoring system in patients with coronary artery disease

**DOI:** 10.1186/s12872-021-01854-5

**Published:** 2021-01-19

**Authors:** Hai-Ping Ma, Lei Zhang, Chun-ling Chen, Jin Li, Zhi Tong Ma, Qiao Qiao Jiang, Yuan Yuan Liang, Shan Shan Li, Fei Long, Hong Zheng

**Affiliations:** Department of Anesthesiology, The First Affiliated Hospital of Xinjiang Medical University, 37 Liyushan South Road, Xinshi District, Urumqi, 830054 Xinjiang China

**Keywords:** Coronary artery disease, Cardiac surgery, Transfusion guidelines, Transfusion trigger score, Red blood cells

## Abstract

**Background:**

A simple and accurate scoring system to guide perioperative blood transfusion in patients with coronary artery disease (CAD) undergoing cardiac surgery is lacking. The trigger point for blood transfusions for these patients may be different from existing transfusion guidelines. This study aimed to evaluate the safety and efficacy of a new scoring strategy for use in guiding transfusion decisions in patients with CAD.

**Methods:**

A multicenter randomized controlled trial was conducted at three third-level grade-A hospitals from January 2015 to May 2018. Data of 254 patients in a Cardiac Peri-Operative Transfusion Trigger Score (cPOTTS) group and 246 patients in a group receiving conventional evaluation of the need for transfusion (conventional group) were analysed. The requirements for transfusion and the per capita consumption of red blood cells (RBCs) were compared between groups.

**Results:**

Baseline characteristics of the two groups were comparable. Logistic regression analyses revealed no significant differences between the two groups in primary outcomes (1-year mortality and perioperative ischemic cardiac events), secondary outcomes (shock, infections, and renal impairment), ICU admission, and ICU stay duration. However, patients in the cPOTTS group had significantly shorter hospital stays, lower hospital costs, lower utilization rate and lower per capita consumption of transfused RBCs than controls. Stratified analyses revealed no significant differences between groups in associations between baseline characteristics and perioperative ischemic cardiac events, except for hemofiltration or dialysis and NYHA class in I.

**Conclusions:**

This novel scoring system offered a practical and straightforward guideline of perioperative blood transfusion in patients with CAD.

*Trial registration* chiCTR1800016561(2017/7/19).

## Background

Cardiovascular disease (CVD) is the number one cause of death globally and has become one of the most important diseases and public health problems in the twenty-first century [1]. The number of patients undergoing cardiac surgery is also increasing yearly, and these patients are more susceptible to the consequences of perioperative anemia [2]. Perioperative anemia in patients with CVD is independently associated with a significantly increased risk of postoperative mortality and major complications [3–7]. Blood transfusion is considered the most effective practice in managing perioperative anemia, which is of critical importance in cardiac surgery because it is directly associated with oxygen supply and delivery [2, 8]. China’s health statistics department reported that the annual cardiac and non-cardiac surgeries in China for CVD-account for 18% of the total surgical volume. Perioperative blood transfusion accounts for 30% of the total amount of blood used, increasing the demand for blood transfusion sufficiently to create a growing gap between supply and demand [9, 10]. At the same time, blood transfusion in the perioperative setting is a risk factor that contributes to perioperative complications, including the transmission of infection, transfusion reactions, altered immune response, circulatory overload, transfusion-related acute lung injury, and increased mortality, ultimately resulting in more extended hospital stays and increased costs [8, 11–13]. From a clinical perspective, strategies to avoid unnecessary blood transfusion and improve patients’ outcomes and overall safety must focus on preventing perioperative anemia; allogenic blood transfusion rates also must be reduced as much as possible to reduce patients’ exposure to the risks of transfusion [11].

The Perioperative Blood Transfusion and Adjuvant Therapy Guide published by the American Association of Anesthesiologists (ASA) in 2006 [14] states that transfusion decisions for patients with hemoglobin (Hb) levels between 6 and 10 g/dL should be based on the objective evaluation of each patient’s clinical condition to determine whether red blood cells (RBC) need to be transfused. Because of this adjustable guideline, there is no evidence‑based evaluation index on when to start blood transfusions in patients with Hb level between 6 and 10 g/dL. As a result, the trigger point for blood transfusion may differ for CVD patients, given the existing transfusion guidelines. Many clinical studies on the threshold for Hb concentrations for RBC transfusions have compared the effects of the liberal transfusion strategy (higher Hb thresholds) and the more restrictive transfusion strategy (lower Hb thresholds) on clinical outcomes [15–19]. Results of such studies have shown that the occurrence of significant complications such as perioperative mortality, septic shock, and myocardial ischemia was not affected by either restrictive or liberal transfusion strategies. Thus, whether or not to use Hb concentration as the transfusion trigger point in cardiac surgical patients is still debatable.

Based on the Peri-operative Transfusion Trigger Score-E (POTTS-E) scheme evaluated by Liu et al. [20], we have proposed and established a new blood transfusion scoring system called Cardiac Peri-Operative Transfusion Trigger Score, abbreviated cPOTTS, that is primarily for patients undergoing cardiac surgery. Our newly developed scoring system is well standardized by considering multiple factors most important in predicting transfusion. It is simple and has better discriminating power than other methods. Hence it is potentially more useful than single-parameter predictors such as Hb concentration alone [20]. The cPOTTS system estimates individual transfusion risks based on patients’ clinical conditions combined with targeted predictors for objective quantification, including requirements for balancing oxygen supply and demand, compensation for cardiopulmonary function, and surgery risks and anesthesia. The present study aimed to evaluate the safety and efficacy of cPOTTS in guiding perioperative blood transfusion in surgical patients with coronary heart disease through a clinical randomized controlled trial, laying the foundation for precise transfusion strategies for this patient population.

## Materials and methods

This work has been reported in line with the Consolidated Standards of Reporting Trials (CONSORT) Guidelines. The clinical trial was registered on the Chinese Clinical Trial Registry (ChiCTR), ID: chiCTR1800016561.

### Patient selection

This multicenter randomized controlled trial was performed at three third-level grade-A hospitals from January 2015 to May 2018. The subjects were included following the diagnostic criteria for coronary artery disease (CAD) reported by the International Society for CVDs and the World Health Organization. After total subjects achieved 500, this trial was terminated without further performing interim analysis.

#### Inclusion criteria

Patient selection was based on the following inclusion criteria: (1) Age ≥ 18 years old and ≤ 75 years old; (2) Permanent residence area < 2500 m above sea level; (3) American Society of Anaesthesiologists (ASA) classification: I–III class; (4) History of angina pectoris; (5) Previous or newly diagnosed ST-segment elevation of more than 1 mm, left bundle branch conduction block or pathological Q wave formation lasting at least 10 min; (6) Positive results of treadmill exercise electrocardiography (ECG); (7) Coronary artery stenoses detected by the multi-detector row CT; (8) Coronary stenosis > 70% or at least primary artery stenosis > 50% clarified by the coronary intervention; (9) Cardiac ultrasound indicating a decrease in ventricular wall movement. Subjects meeting the criteria 1, 2, 3 combined with one of criteria 4, 5, 6, 7, 8, 9 were included in this study.

#### Exclusion criteria

Patients with any of the following criteria were excluded: (1) emergency surgery; (2) clinical diagnosis of severe blood system diseases; (3) clinical diagnosis of a defect in the oxygen-carrying capacity of hemoglobin; (4) tumor metastasis; (5) inability, for any reason, to cooperate with the study (for example, poor language comprehension, mental illness, and receiving any other drugs or participation in any other clinical trials within 3 months before the selected research; (6) researchers did not consider it appropriate for patients to be included in this study.

### Coronary Peri-Operative Transfusion Trigger Score, cPOTTS

#### The cPOTTS marking strategy

All patients’ transfusion requirements and the per capita consumption of RBCs were evaluated and compared following the cPOTTS marking strategy of the First Affiliated Hospital of Xinjiang Medical University. As shown in Table 1, circulation was evaluated by determining whether patients needed adrenaline to maintain normal blood pressure (0–2 points); blood oxygenation situations were evaluated by determining whether higher peripheral capillary (SpO_2_) (0–2 points) and central venous (ScvO_2_) (0–2 points) oxygen concentrations were needed to maintain oxygenation; heart conditions were evaluated by the incidence of angina pectoris during certain physical conditions (0–2 points) and whether ST segment was down ≥ 0.1 mV or arrhythmia occurred during surgery (2 points).

**Table 1:** Coronary Peri-Operative Transfusion Trigger Score (cPOTTS)

Score	Epinephrine (μg kg^−1^ min^−1^)^a^	FiO_2_ (%)^b^	ScvO_2_^c^	Angina pectoris
0	0	≤ 35%	> 75%	No
+ 1	0–0.05	36–50%	70–75%	Occurs during exercise or manual labor or excitement
+ 2	≥ 0.06	≥ 51%	< 70%	Occurs during daily activity or quietly rest

During the operation, if ST segment was down ≥ 0.1 mV and or arrhythmia occurred, it was scored at 2 points. The total scores of four above items + 7 were the total score of cPOTTS. The top score was 10 points, and atotal score ≥ 10 was still scored as 10 points

^a^The amount of epinephrine needed to maintain normal blood pressure

^b^Requirement of inspiration O_2_ to sustain 95% SpO_2_ (peripheral capillary oxygen saturation)

^c^Central venous oxygen saturation

In the non-cardiac surgery cases, the “angina pectoris” column in the cPOTTS chart was scored according to the incidence of angina pectoris within 6 months before surgery. In principle, the preoperative score for this item was typical throughout the hospital stay. If angina pectoris was confirmed after the operation or was aggravated, the score was increased; however, when angina pectoris was relieved, the score was not reduced.

Patients with angina pectoris and diagnosed with coronary heart disease were treated with routine medication, and most of them underwent coronary artery bypass surgery. If the subject underwent coronary artery bypass grafting, the score in the “angina pectoris” column in the cPOTTS chart must be divided into two stages: preoperative and postoperative.

Before surgery, patients’ transfusion risks were scored according to the incidence of angina within 6 months before surgery and used to guide blood transfusion decisions to maintain Hb levels between the time of admission to the beginning of extracorporeal circulation (or non-extracorporeal circulation bypass surgery) and between the beginning of extracorporeal circulation (or non-extracorporeal circulation bypass surgery) until the patient returned to consciousness after surgery. During the postoperative consciousness period to hospital discharge, the score in the “angina pectoris” column in the cPOTTS chart was measured and used as a guide for RBC transfusion.

For all patients in the cPOTTS group, the baseline trigger point was 7, cPOTTS was 0–10 points, and blood transfusion indications were 7 + cPOTTS scores (total score). If the total score was ≥ 10 points, RBC infusion was performed according to the total score at 10 points. Subjects were scored according to cPOTTS marking strategy and Hb values, and blood transfusion was initiated based on the total score.

#### Blood transfusion guidelines

According to the transfusion guidelines reported by the American Society of Anesthesiologists Task Force on Perioperative Blood Transfusion and Adjuvant Therapies [14], patients with a Hb value of 7–10 g/dL require a blood transfusion. For patients with Hb ≥ 7 g/dL (7 points), ≥ 8 g/dL (8 points), ≥ 9 g/dL (9 points) and ≥ 10 g/dL (10 points) had no need for allogeneic RBC transfusion according to this guidelines. If a patient’s Hb was still less than these levels after all collected autologous blood had been returned, allogeneic RBC transfusion should be performed. However, after the allogeneic RBC transfusion, patients’ Hb should be maintained at the same levels described in the above scoring groups.

#### Methods of cPOTTS implementation

The cPOTTS score is a dynamic score equal to the score at the time specified in the case report form (CRF) table and before each preparation for allogeneic RBC transfusion. The score is also a real-time score based on when the blood volume was within normal limits, and blood volume was determined to be expected based primarily on the physician’s clinical evaluation. The study subjects did not undergo acute hypervolemic hemodilution during the perioperative period. Whether the patient’s cardiac output was expected was also determined mainly by the physician based on clinical indicators such as blood pressure, heart rate, and urinary output (we did not use transesophageal echocardiography because it is invasive). The central venous pressure (CVP) at the time of evaluation and the results of various cardiac output measurements can also be used as important references. Clinical evaluation of postoperative cardiac output continued to show it was lower than average. Only continuous pumping of adrenaline was used to increase cardiac output. The cPOTTS scoring process considered the adrenaline infusion rate required to maintain a clinically assessed expected cardiac output. Other factors, such as transient bradycardia and hyperpiesia, acute blood loss requiring the administration of other cardiovascular active drugs (e.g., atropine, ephedrine, dopamine, m-hydroxylamine, norepinephrine, phenylephrine, etc.) were not included as cPOTTS score items. Methods using the fraction of inspiration O_2_ parameters to maintain the SpO_2_ ≥ 95% over continuous inhalation time of at least 10 min are described as follows: (1) Patients received oxygen from the nasal cannula at the oxygen flow rate of 3.5 L/min with a fraction of inspiration O_2_ (FiO_2_%) of 35% or from the oxygen mask at fresh oxygen flow ≥ 6 L/min; FiO_2_% value also considered at ≥ 50%. (2) When mechanical ventilation was performed by the ventilator, the FiO_2_% value was determined according to the ventilator parameters. (3) When the mechanical ventilation was performed by the anesthesia machine, FiO_2_% value was 35% at an oxygen flow rate of 1.8 L/min, and airflow rate of 8.2 L/min or oxygen flow rate of 3.5 L/min, and nitrous oxide flow rate of 6.5 L/min; and FiO_2_% value was 50% at an oxygen flow rate of 3.7 L/min and an airflow rate of 6.3/min or at an oxygen flow rate of 5.0/min and nitrous oxide flow rate of 5.0 L/min. It was recommended that arterial blood gases (ABG) should be measured to determine the SaO_2_ (oxygen saturation) value when the clinical SpO_2_ was not possible or unable to be measured accurately. For central venous oxygen saturation, ScvO_2_ was measured by deep venous (intracranial vein or subclavian vein) blood gas test.

The “angina pectoris” column in the cPOTTS chart was scored preoperatively according to the incidence of angina pectoris within 6 months before surgery. During the operation, the “angina pectoris” column in the cPOTTS chart was scored based on ECG results: an ECG showing ST-segment depression ≥ 0.1 mV and or arrhythmia scored 2 points; if there were no changes in ECG, the score was based on preoperative angina. If postoperative evaluation confirmed that the angina was worse, 1 point was added; if the angina were relieved, the score would not change.

### Blood transfusion for the conventional transfusion group

According to guidelines for blood transfusion formulated by the Ministry of Health of the People’s Republic of China in 2000 [21], when a patient’s Hb was > 10 g/dL, RBC transfusion is not required. When a patient’s Hb is < 7 g/dL, transfusion should be considered. When a patient’s Hb is between 7 and 10 g/dL, RBC transfusion should be considered according to patients’ cardiopulmonary compensatory function, body metabolism, and oxygen consumption. According to blood transfusion guidelines, the anesthesiologist and/or the surgeon in charge should judge the time and amount of RBC transfusion subjectively.

### Randomization procedure and sample size calculation

This study's subjects were randomly assigned to the cPOTTS group and the conventional group described below according to the computer randomization software (SAS 9.1). Grouping results were loaded into opaque envelopes and distributed to the three participating centers. A 5-digit randomization sequence was written on the envelope's cover. According to the time within which the subjects were selected, each center disassembled envelopes from low to high randomized numbers on the cover of the envelope, which determined random assignment of study subjects. To avoid bias, the operators and evaluators were different physicians.

The sample size was calculated based on the following equation by PEMS3.1 software:

$$n = 2\lambda /\left( {2\sin^{ - 1} \sqrt {P_{\max } } - 2\sin^{ - 1} \sqrt {P_{\min } } } \right)^{2}$$

when α = 0.05 and β = 0.1, at least 510 patients should be enrolled in each group. The interim analysis was omitted because the trial was closed when we collected as many as 500 cases as expected.

### Measurements

Primary outcomes were all-cause mortality at 30 days and 1-year after surgery; perioperative ischemic cardiac events (myocardial ischemia, myocardial ischemia, myocardial infarction, heart failure); secondary outcomes were shocking; the incidence of lung, digestive system and incisional infections; and renal impairment. The observation index included changes in hemoglobin levels, ICU admission, ICU stay duration, hospital stay duration and hospital costs.

### Statistical analysis

For patient's descriptive statistics, categorical variables are reported as number (n) and percentage (%); continuous variables are reported as mean ± standard deviation (SD), and the total volume of RBC transfusion is reported as median and interquartile range. An independent *t* test was used to compare data between the two groups. Logistic regression analyses were performed to identify associations between the two groups in outcomes and observation indexes. Subgroup analyses were performed to explore the potential risk factors for postoperative perioperative cardiac ischemia (a *p* value of less than 0.05 was accepted) between two groups. *p* < 0.05 (2-sided) was considered to be statistically significant. All statistical analyses were performed using the statistical software package SPSS complex sample module version 22.0 (IBM Corp, Armonk, NY, USA).

## Results

### Study population

**Fig. 1:**
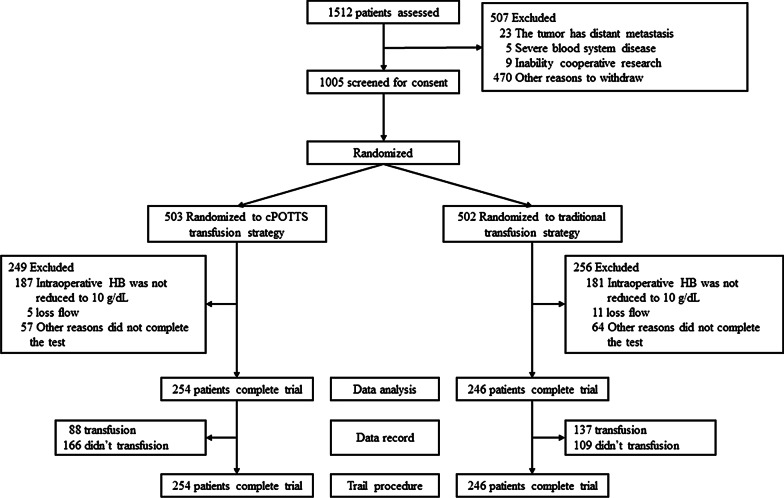
Study flow

A total of 1512 patients were assessed for eligibility (Fig. 1). After exclusions for various medical and other reasons, 1005 patients were enrolled in the study: 503 were assigned to the cPOTTS group and 502 to the conventional group. Of these, 505 patients (50.2%) (249 in the cPOTTS group and 256 in the traditional group) were further excluded after intraoperative Hb was not reduced to 10 g/dL, loss of blood flow or other reasons resulted in not completing the trial, leaving 500 patients (254 in the cPOTTS group and 246 in the conventional group) as the final analytic sample.

As is shown in Table 2, baseline characteristics were well balanced between the study groups. The mean age of the cPOTTS group was 63.3 ± 10.8 years, while the mean age of the conventional group was 64.4 ± 10.6 years. Most patients were male (65% and 63% in the cPOTTS and conventional group, respectively), with NYHA class П (47.6% and 47.2% in the cPOTTS and conventional group, respectively), were without investigation of vessels regarding CAD, had hypertension, used aprotinin and were without previous cardiac surgery. Hemoglobin concentrations at the time of randomization were similar in the cPOTTS and conventional groups (mean 114.99 ± 16.55 g/dL vs 114.02 ± 19.99 g/dL, *p* = 0.555). No significant differences were noted between the two groups regarding baseline characteristics, except for hemofiltration or dialysis, and NYHA class in I, significantly higher in the conventional group (Table 2).

**Table 2:** Baseline characteristic of study population

Variables, n (%)	cPOTTS group n = 254	Conventional group n = 246	*p* value
Demographic characteristics			
Age, mean ± SD	63.3 ± 10.8	64.4 ± 10.6	0.246
Male	165 (65.0)	155 (63.0)	0.649
Body mass index, mean ± SD	25.20 ± 3.87	25.44 ± 3.81	0.475
NYHA class			0.059
I	39 (15.4)	22 (8.9)	**0.029**
П	121 (47.6)	116 (47.2)	0.914
Ш	84 (33.1)	102 (41.5)	0.052
IV	10 (3.9)	6 (2.4)	0.354
Echocardiogram	254	245	
Ejection fraction (%)	56.7 ± 3.3	57.1 ± 3.6	0.564
Coronary artery disease			0.383
Single-vessel	13 (5.1)	6 (2.4)	0.117
Double-vessel	41 (16.1)	41 (16.7)	0.874
Triple-vessel	84 (33.1)	76 (30.9)	0.602
Not investigated	116 (45.7)	123 (50.0)	0.332
Combined diseases			
Diabetes	82 (32.3)	68 (27.6)	0.258
Hypertension	139 (54.7)	135 (54.9)	0.972
COPD	3 (1.2)	6 (2.4)	0.332
Hemofiltration or dialysis	0	5 (2.0)	**0.028**
Cerebrovascular accident /transient ischemic attack	1 (0.4)	3 (1.2)	0.366
Preoperative Hemoglobin g/L, mean ± SD	114.99 ± 16.55	114.02 ± 19.99	0.555
Drugs taken			
β-Receptor blocker	45 (17.7)	53 (21.5)	0.281
ACEI	37 (14.6)	32 (13.0)	0.613
Calcium antagonist	28 (11.0%)	36 (14.6)	0.227
Aprotinin	119 (46.9)	117 (47.6)	0.874
Others	39 (15.4)	30 (12.2)	0.306
Type of surgery			0.379
Cardiac surgery (Coronary artery bypass graft)	110 (43.3)	97 (39.4)	
Non-cardiac surgery	144 (56.7)	149 (60.6)	
Hepatobiliary surgery	37 (26.4)	32 (21.5)	0.613
Gastrointestinal surgery	48 (30.7)	50 (33.6)	0.688
Gynecological surgery	39 (23.2)	36 (24.2)	0.822
Urinary surgery	20 (19.7)	31 (20.1)	0.081

Bold indicates statistically significant difference

*cPOTTS* Cardiac Peri-Operative Transfusion Trigger score, *NYHA* New York Heart Association, *COPD* Chronic obstructive pulmonary disease

### Transfusion intervention

Blood loss and transfusions in the two groups are shown in Table 3. The mean intraoperative blood loss of patients in the cPOTTS group was 395.77 ± 367.68 mL and was 476.30 ± 385.20 mL in the conventional group. Patients in the conventional group received blood transfusions than those in the cPOTTS group (n = 68; 26.8% vs n = 134; 54%, p = 0.017). The conventional and cPOTTS groups received a median of 3 RBC units for each patient (IQR 2–5 vs IQR 2–4, Mann–Whitney *t* test p = 0.337). The number of patients receiving 1–4 units of RBC transfusion in the cPOTTS group was lower than that in the conventional group. Plasma transfusion was given to 61 (24.0%) patients in the cPOTTS group and 89 (36.2%) patients in the conventional group (Table 3).

**Table 3:** Comparison of blood loss and RBC transfusions in cPOTTS and conventional groups

Variables	cPOTTS group n = 254	Traditional group n = 246	*p* value
Intraoperative blood loss (ml), mean ± SD	395.77 ± 367.68	476.30 ± 385.20	**0.017**
RBC transfusion, n (%)	68 (26.8)	134 (54.5)	**< 0.001**
Total RBC transfusion (U)			
Median (Q1, Q3)	3 (2, 4)	3 (2, 5)	0.337
Distribution-no. of patient (%)			**< 0.001**
0-Units	186 (73.2%)	112 (45.5%)	**< 0.001**
1-Units	3 (1.2%)	6 (2.5%)	0.471
2-Units	23 (9.1%)	49 (19.9%)	**< 0.001**
3-Units	22 (8.7%)	50 (20.4%)	**< 0.001**
4-Units	10 (3.8%)	17 (6.9%)	0.141
5 ≥ Units	10 (4%)	12 (4.8%)	0.608
Other transfusion			
Plasma	61 (24%)	89 (36.2%)	**0.003**
Platelets	0	1 (0.4%)	NA
Cryoprecipitate	0	2 (0.8%)	NA

Bold indicates statistically significant difference

*cPOTTS* Cardiac Peri-Operative Transfusion Trigger score, *RBCs* red blood cells

No significant differences were found in preoperative or postoperative hemoglobin concentrations between patients in the cPOTTS group and those in the conventional group (Table 4). Hemoglobin concentrations in two groups were slightly lower at peri-operation than post-operation, but with no significant differences.

**Table 4:** Comparison of perioperative and postoperative haemoglobin concentrations between groups

Variables, mean ± SD	cPOTTS group n = 254	Conventional group n = 246	*p* value
Perioperative Hb (g/dL)			
Admission to Hb (g/dL)	116.71 ± 33.78	116.52 ± 3.61	0.984
Into operating room Hb (g/dL)	114.99 ± 16.55	114.02 ± 19.99	0.555
End of operation Hb (g/dL)	96.63 ± 11.37	97.49 ± 12.13	0.414
Postoperative Hb (g/dL)			
Postoperative 24 h Hb (g/dL)	108.40 ± 15.84	107.60 ± 17.23	0.587
Before discharge 48–72 h Hb (g/dL)	109.15 ± 16.47	106.48 ± 17.11	0.077
Before discharge 24 h Hb (g/dL)	108.24 ± 17.28	106.29 ± 16.31	0.197

Bold indicates significant difference (*p* < 0.05)

cPOTTS: Cardiac Peri-Operative Transfusion Trigger score, *Hb* haemoglobin, *ICU* intensive care unit

### Outcome measures

Logistic regression analyses revealed no statistical differences between the two groups in primary outcomes, including 1-year mortality and ischemic cardiac events (myocardial ischemia, myocardial infarction, arrhythmia, heart failure); secondary outcomes, including shock, infections (incision infection, digestive system infection, lung infection); renal impairment; and observation index, including ICU admission and duration of ICU stay. However, patients in the conventional group had significantly longer hospital stays and higher hospital costs than those in the cPOTTS group [β = 1.78, 95% confidence intervals (CI) 0.03–3.53, *p* = 0.04; β = 1.66, 95% CI 0.50, 2.82, *p* = 0.01, respectively] (Table 5).

**Table 5:** Primary and secondary outcomes and observation indexes

	cPOTTS group	Conventional group	β coefficient	*p* value
Primary outcomes				
All-cause mortality				
1-year mortality	0	2 (0.81%)	–	–
Ischemic cardiac event				
Myocardial ischemia	32 (12.6%)	20 (8.13%)	0.61 (0.34–1.11)	0.10
Myocardial infarction	13 (5.12%)	8 (3.25%)	0.62 (0.25–1.53)	0.30
Arrhythmia	1 (0.39%)	3 (1.22%)	3.12 (0.32–30.23)	0.33
Heart failure	1 (0.39%)	4 (1.63%)	4.18 (0.46–37.68)	0.20
Secondary outcomes				
Shock	17 (6.69%)	12 (4.88%)	0.72 (0.33–1.53)	0.39
Infection	18 (7.09%)	8 (3.25%)	0.44 (0.19–1.03)	0.06
Incision infection	10 (3.94%)	3 (1.22%)	0.30 (0.08–1.11)	0.07
Digestive system infection	1 (0.39%)	1 (0.41%)	1.03 (0.06–16.60)	0.98
Lung infection	7 (2.76%)	4 (1.6%)	0.58 (0.17–2.02)	0.40
Renal impairment	5 (1.97%)	2 (0.81%)	0.41 (0.08–2.12)	0.29
Observation index				
ICU admission [case (%)]	182 (71.65%)	193 (78.46%)	1.44 (0.96–2.17)	0.08
Duration of ICU stay (day)	1.53 ± 2.08	1.79 ± 2.66	0.26 (− 0.16, 0.68)	0.23
Duration of hospital time (day)	19.76 ± 7.26	21.54 ± 12.11	1.78 (0.03, 3.53)	**0.04**
Hospital costs (¥: Ten thousand)	9.05 ± 4.32	10.70 ± 8.33	1.66 (0.50, 2.82)	**0.01**

**Fig. 2:**
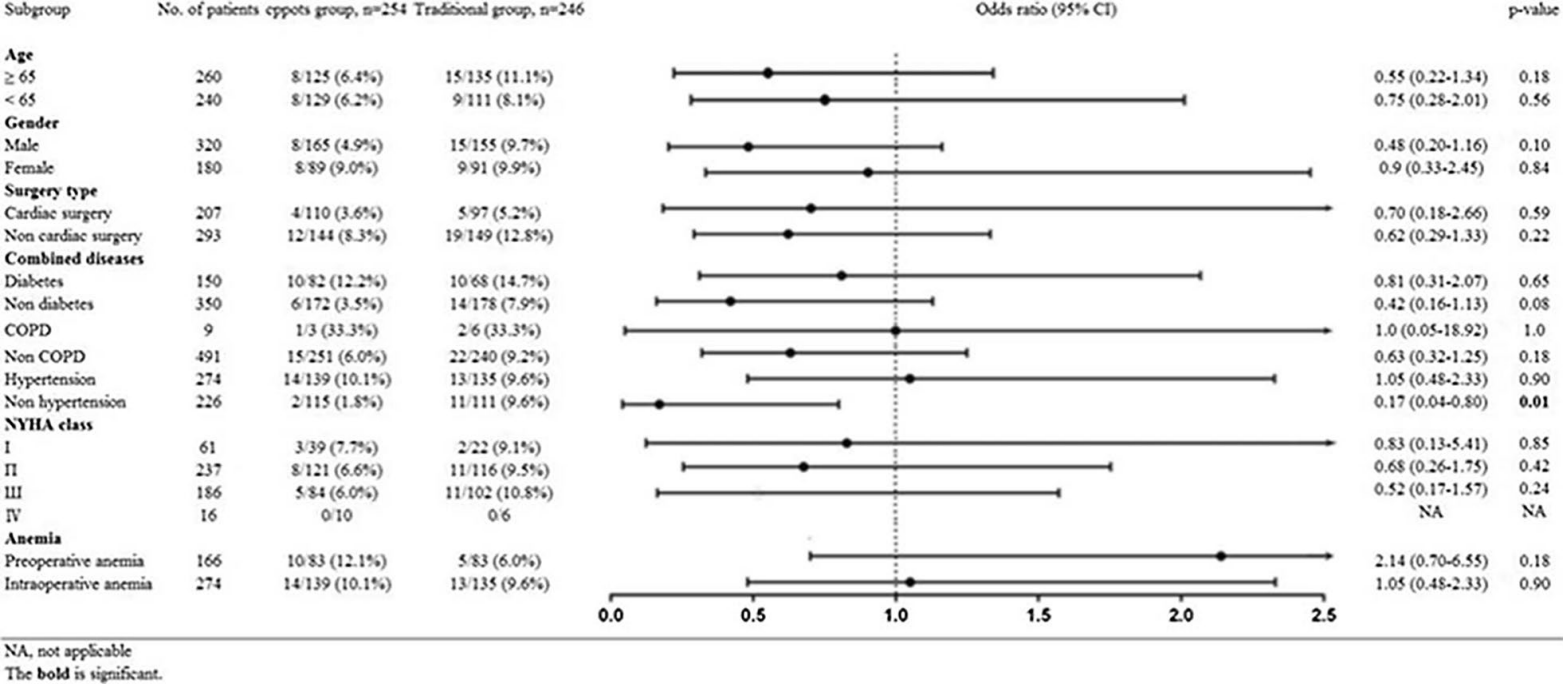
Subgroup analysis of patient characteristics for perioperative ischemic cardiac events between groups

Subgroup analyses of perioperative ischemic cardiac events in the two groups are presented in Fig. 2. In the non-hypertension subgroup, patients in the cPOTTS group had significantly lower odds ratios in perioperative ischemic cardiac events than those in the conventional group (OR = 0.17, 95% CI 0.04–0.80, *p* = 0.012). However, no significant differences were found between the two groups in other subgroups (Fig. 2).

## Discussion

Our cardiovascular team developed a scoring system that predicts the likelihood of transfusion in patients with perioperative anemia during cardiac surgery. In this randomized controlled trial, we compared the newly designed cPOTTS scoring system to the conventional method for determining whether or not transfusion is needed. Characteristics of the two groups of included patients were consistent at the preoperative baseline, and all data were comparable. The new cPOTTS scoring system achieved similar primary and secondary outcomes as the conventional method. However, the utilization rate and the per capita consumption of RBC were both higher in the conventional group than in cPOTTS group. We also found that patients using the cPOTTS scoring system had significantly shorter hospital stays and lower hospital costs than those using the conventional method. Stratified analyses showed that patients with non-hypertension using the cPOTTS scoring system had a significantly lower odds ratio in perioperative ischemic cardiac events than those using the conventional method.

RBC transfusion is a significant factor in cardiac surgery and is known to increase morbidity and mortality [22]. The increased demand for RBC availability for cardiac surgery is also an economic burden [23]. As an organ with very high oxygen and energy requirements, the myocardium extracts approximately 75% of the oxygen supplied by coronary blood flow at rest and ensures sufficient blood supply and oxygen supply [24]. Patients with CAD have unique pathophysiological characteristics, including different stenosis degrees in the coronary arteries, which induces inefficient blood and oxygen supply to the myocardium. Therefore, when a critical condition occurs, there is almost no reserve for increased myocardial oxygen consumption. In patients with coronary artery disease, the compensation mechanism for delivering maximal oxygen to the tissue during perioperative anemia is impaired [25]. Therefore, perioperative blood transfusions are a very complex issue for patients with CAD.

The conventional method of identifying the trigger point for transfusion is based on an anesthesiologist or surgeon's experience or judgment to decide whether a particular patient needs RBC transfusion [26]. Therefore, a certain gap may exist between the patient's situation and the physician's experience, leading to blood transfusion abuse and waste of medical resources. Due to inappropriate blood transfusions, the incidence and of CAD patients’ perioperative cardiac adverse events are significantly increased [27, 28]. In addition, practical guidelines regarding the effectiveness of red blood cell transfusion strategy for patients who underwent cardiac surgery and were at moderate-to-high risk for death are limited [29]. In the present study, we designed a new cPOTTS scoring system to replace the required subjective judgment in conventional blood transfusion indications when patients’ Hb is 7–10 g/dL. The cPOTTS scoring system is based on the principles of oxygen supply and demand balance, representative of cardiac function (epinephrine use), lung function (maintaining SPO_2_ ≧ 95% required inhaled oxygen concentration), oxygen consumption (ScvO_2_), and myocardial ischemia (angina pectoris). These factors are used to evaluate the body’s oxygen supply and demand balance and objectively assess the essential oxygen supply and demand levels. As such, the cPOTTS scoring system strives to achieve accurate and individualized blood transfusion according to the functional state of an individual’s organs.

In the present study, we observed no statistically significant differences between the cPOTTS-guided transfusion strategy and the guidelines in mortality and ischemic cardiac ischemic cardiac complications (myocardial ischemia, myocardial infarction, arrhythmia, and heart failure), as well as shock, infection, and renal impairment. However, the number of cPOTTS group patients receiving 2–4 units of RBC transfusion was lower than that in the conventional group, which reduced the demand for RBC availability for cardiac surgery and alleviated a potential economic burden. The cPOTTS-guided transfusion strategy reduces the number of patients receiving small or moderate blood transfusions without increasing the risk of myocardial ischemia-related complications. The stratified analysis also indicated no significant difference in the incidence of ischemic cardiac events between the two groups in different subgroups such as age, type of surgery (cardiac and non-cardiac surgery), and different cardiac functions, indicating that the cPOTTS-guided transfusion strategy has widespread applicability.

The primary purpose of perioperative blood transfusion is to restore hemoglobin and hematocrit levels quickly and effectively [30, 31], but the safety and effectiveness of perioperative transfusion are still in doubt. Some studies have reported substantial adverse effects of blood transfusions on patients’ short- and long-term prognosis [15, 32]. We observed no statistically significant differences between groups in perioperative cardiac adverse events (n = 47; 18.5% vs n = 35; 14.2%), indicating that the cPOTTS blood transfusion scoring system not only reduced the utilization rate and the per capita consumption of blood transfusion but also considered the balance of oxygen supply and demand. Also, the hospital costs were significantly lower in patients using the cPOTTS scoring system than those using the conventional method, which may be due in part to the decrease in blood transfusion-related costs.

Recent systematic reviews have confirmed that there are no significant differences in factors associated with mortality and myocardial infarction between restrictive or the liberal transfusion strategies [15, 26]. However, the evidence's overall quality is not sufficient [32]. Hebert et al. [15] suggested that restrictive transfusion strategies are not associated with an increase in adverse events (mortality, cardiac events, stroke, pneumonia, and thromboembolism) compared with liberal transfusion strategies; hence, restrictive transfusion strategies can reduce hospital mortality, but not 30-day mortality. Hajjar et al. [26] also reported that using a restrictive perioperative transfusion strategy in patients undergoing cardiac surgery had similar results as the combined outcome of 30-day all-cause mortality and severe morbidity compared with the liberal strategy. However, a review published in 2014 showed that hospital mortality, total mortality, pulmonary edema, and bacterial infection were reduced using a restricted hemoglobin transfusion trigger point of 7 g/dL, compared with a liberal transfusion strategy [33].

In the present study, the cPOTTS scoring system’s implementation as the real-time dynamic scoring method was designed to qualify the targeted Hb value of blood transfusion while considering the trigger point of Hb value belonging to the restrictive blood transfusion strategy. Therefore, cPOTTS avoids the limitation of restrictive blood transfusion strategy, saving the amount of blood transferred, reduces the risk of blood transfusion-related complications, and achieves the purpose of accurate blood transfusion management.

Perioperative ischemic cardiac events are the most important clinical outcome in patients with CAD. In the present study, stratified analyses of patients of different age, sex, type of operation, cardiac function classification, and other types of perioperative myocardial ischemic events than the two blood transfusion strategies in perioperative ischemic cardiac events were not statistically different. Patients with non-hypertension using the cPOTTS scoring system had a significantly lower odds ratio in perioperative ischemic cardiac events compared to those using the conventional method. Overall, we validated that the cPOTTS scoring system has possible applicability in the clinical setting.

The present study has several limitations, including the limited sample size. There were no restrictions on the type of anesthesia used for patients, and minor effects of anesthesia on oxygen supply and demand may be a confounding factor. Also, because the cPOTTS scoring system strategy involves more strict control of Hb estimations, discrepancies may occur between a patient’s actual Hb and the estimated value after blood transfusion. Both cardiac surgery and non-cardiac surgery were included in this study; thus, there may be some differences in the amount of blood loss and the choice of blood transfusion. The new cPOTTS scoring system has only been practiced in these three specific hospitals in this study, which may not accurately reflect general transfusion practice in all centers. Therefore, the system would require recalibration if applied to other centers. A simple App/online system to estimate cPOTTS scores will be designed and implemented in the future in order to introduce this scoring system for carrying out multicenter and large sample clinical studies.

We also should mention that around 50% of participants were excluded from this study. The reasons could be intraoperative Hb of patients higher than 10 g/dL, blood flow loss of patients, or the exclusion from a subjective decision of researchers, causing the high exclusion rate; however, control was difficult because of the divergent responses for each patient. Our exclusion criteria did not exclude patients using antiaggregant and anticoagulant therapies before this research. The antiaggregant can facilitate bleedings and might be an interference in this transfusion study. Besides, 50% and 45.7% of the conventional and cPOTTS group did not have a coronary assessment, respectively, which might contain limitations. However, considering that each group's demographic and clinical data were comparable, the study data still suggest the advantages of cPOTTS.

## Conclusions

In this study, using the Cardiac Peri‑operative Transfusion Trigger Score evaluation strategy to guide the application of RBC transfusion in patients with coronary artery disease demonstrated better or equal performance with conventional transfusion guidelines. Compared with surgeon and/or anesthesiologist experience‑based subjective assessment, this scoring strategy was closer to patients’ physiological needs for transfusion, reducing perioperative complications, utilization rates and per capita consumption of RBC. Improvement was seen in the prognosis and outcomes of patients, and medical costs declined. Therefore, cPOTTS scores could be a practical and feasible strategy for individualizing perioperative blood transfusion in patients with coronary artery disease. Besides, more large patients studies could be performed to prove the safety of ePOTTS system.

## Data Availability

The datasets analysed during the current study are available from the corresponding author on reasonable request.

## Abbreviations

CAD: Coronary artery disease
cPOTTS: Cardiac Peri-Operative Transfusion Trigger Score
CVP: Central venous pressure
